# Examining the Association Between Psychopathy Clusters and Risk-Taking Behaviors

**DOI:** 10.1192/j.eurpsy.2024.283

**Published:** 2024-08-27

**Authors:** R. Gómez Leal, P. Fernández-Berrocal, A. Megías-Robles

**Affiliations:** University of Málaga, Málaga, Spain

## Abstract

**Introduction:**

Psychopathy encompasses the sub-dimensions of interpersonal manipulation, callous affect, erratic lifestyle, and criminal tendencies. Most studies investigating this trait have traditionally utilized a variable-centered approach. However, in the current study, we have adopted a person-centered approach.

**Objectives:**

Our objective was to analyze distinct homogeneous subgroups of individuals characterized by specific psychopathy profiles and examine their relationship with risk-taking behavior.

**Methods:**

Our sample consisted of 371 participants (26.4% men, aged 18 to 59 years), who completed the 34-item Self-Report Psychopathy Scale-III to assess psychopathy and Risk-taking behaviors were assessed using the Domain-Specific Risk-Taking Scale (DOSPERT-30).

**Results:**

Through cluster analysis, we identified four distinct groups: Low psychopathy, Low criminal tendencies, High erratic lifestyle, and High psychopathy group. The primary findings revealed that the High psychopathy group, characterized by elevated scores in all sub-dimensions, exhibited higher levels of Risk-Taking Behaviors and a lower Perception of Risk compared to the other groups. Furthermore, the Low criminal tendencies group, marked by high scores in all dimensions and low scores in criminal tendencies, demonstrated greater risk-taking behavior compared to the Low psychopathy and High erratic lifestyle groups.

**Conclusions:**

These results stimulate the debate about whether it is appropriate to incorporate the dimension of criminal tendencies within the concept of psychopathy. Certain clinical implications emerge from this study that are deserving of a comprehensive and thoughtful discussion.

**Disclosure of Interest:**

None Declared

